# Review of the South American leafhopper genus *Parandanus* (Hemiptera, Cicadellidae, Deltocephalinae)

**DOI:** 10.3897/zookeys.562.7478

**Published:** 2016-02-10

**Authors:** Yani Duan, Christopher H. Dietrich, Micael D. Webb, Yalin Zhang

**Affiliations:** 1 School of Plant Protection, Anhui Agricultural University, Hefei, Anhui Province 230036, China; 2 Illinois Natural History Survey, Prairie Research Institute, University of Illinois at Urbana-Champaign, 1816 S. Oak St., Champaign, IL 61820, USA; 3 The Natural History Museum, London, UK, SW7 5BD; 4 Key Laboratory of Plant Protection Resources and Pest Management of the Ministry of Education, Entomological Museum, Northwest A & F University, Yangling, Shaanxi Province 712100, China

**Keywords:** Auchenorrhyncha, morphology, taxonomy, new species

## Abstract

The South American leafhopper genus *Parandanus* Linnavuori & DeLong (Deltocephalinae: Deltocephalini) is reviewed and four of its six species are illustrated and male genital characters are provided. Three new species from Peru, *Parandanus longistylus* Duan, **sp. n.**, *Parandanus nigricephalus* Duan, **sp. n.** and *Parandanus paracruciatus* Duan, **sp. n.** are described. A key to species is also provided.

## Introduction

The South American grassland leafhopper genus *Parandanus* Linnavuori & DeLong (Deltocephalinae: Deltocephalini) was established by Linnavuori and DeLong (1976) for two species from Peru, *Parandanus hilaris* and *Parandanus ornatus* (the type species). Later, Linnavuori and DeLong (1979) described *Parandanus cruciatus* from Bolivia. Zahniser and Dietrich (2013) included the genus in tribe Deltocephalini based on the linear connective fused to the aedeagus. Members of this genus can be recognised by the following combination of characters: frontoclypeus with pale median longitudinal stripe, anal tube membranous and aedeagus with pair of basal or subbasal appendages. In this paper, *Parandanus* are reviewed with description of three new species from Peru. All species of the genus are listed and a key to males is provided.

## Materials and methods

The material studied here is deposited in the Illinois Natural History Survey (INHS). Morphological terminology follows Zhang (1990) and Dietrich (2005). Digital photographs were taken with a QImaging Micropublisher 3.3 digital camera mounted on an Olympus BX41 stereo microscope and with a Nikon D1x digital SLR camera configured with lenses by Microptics, Digital Lab XLT system. Photographs were modified with Adobe Photoshop CS.

## Taxonomy

### Parandanus

Taxon classificationAnimaliaHemipteraCicadellidae

Linnavuori & DeLong

Parandanus Linnavuori & DeLong, 1976: 34. Type species: *Parandanus ornatus* Linnavuori & DeLong, 1976.

#### Redescription.

Overall coloration pale yellow with orange to dark brown markings. Frontoclypeus with pale median longitudinal stripe and indistinct pale lateral arcs. Pronotum with six longitudinal bands. Scutellum with basal triangles and a medial stripe, orange to sordid brown. Forewing veins pale, bordered with fuscous. Mesosternum dark brown. Femora and tibiae with fuscous marks.

Body elongate. Head narrower than pronotum. Crown not or only slightly produced, rounded to face; ocelli next to eyes on anterior margin. Face relatively flat, similar in width to length; frontoclypeus relatively narrow; clypeal sulcus absent; anteclypeus nearly parallel-sided, extended to ventral margin of face; genae broad, insinuated near eyes. Forewing long and narrow, appendix distinct, with four apical and three anteapical cells, inner anteapical cell open basally. Anal tube membranous.

#### Male genitalia.

Pygofer without processes, with numerous macrosetae in posterior region. Subgenital plate elongate triangular, with few stout to many more slender macrosetae laterally. Style with articulating arm short to very long; apophysis short to long. Connective fused to aedeagus, arms close to each other. Aedeagal shaft slender with pair of basal or subbasal elongate appendages extended posteroventrally along shaft; gonopore apical on dorsal surface.

#### Distribution.

Bolivia, Peru.

#### Checklist of species of *Parandanus*

*Parandanus cruciatus* Linnavuori & DeLong, 1979. Bolivia.

*Parandanus hilaris* Linnavuori & DeLong, 1976. Peru.

*Parandanus longistylus* Duan, **sp. n.** Peru.

*Parandanus nigricephalus* Duan, **sp. n.** Peru.

*Parandanus ornatus* Linnavuori & DeLong, 1976. Peru.

*Parandanus paracruciatus* Duan, **sp. n.** Peru.

#### Key to species of *Parandanus* (males)

**Table:** 

1	Style preapical angle indistinct; apophysis long (Fig. 2D)	**2**
–	Style preapical angle distinct; apophysis short (Fig. 4D)	**3**
2	Style >4× longer than distance between lobes of apodeme (Fig. 2D). Aedeagal appendages terminating well short of aedeagal shaft apex (Fig. 2E–G)	***Parandanus longestyle* Duan, sp. n.**
–	Style <3× longer than distance between lobes of apodeme (Fig. 6D). Aedeagal appendages terminating near aedeagal shaft apex (Fig. 6E–G)	***Parandanus nigricephalus* Duan, sp. n.**
3	Aedeagal appendages not crossing each other ventrally (Linnavuori and DeLong 1976: Fig. 94).	***Parandanus ornatus* Linnavuori & DeLong**
–	Aedeagal appendages crossing each other ventrally (Fig. 4E–F)	**4**
4	Subgenital plate with lateral margins distinctly emarginate near base (Fig. 8D)	***Parandanus hilaris* Linnavuori & DeLong**
–	Subgenital plate with lateral margins nearly straight throughout length (Fig. 4C).	**5**
5	Aedeagal appendages subbasal and curved posterodorsad (Fig. 4E–F)	***Parandanus paracruciatus* Duan, sp. n.**
–	Aedeagal appendages basal and directed posteroventrad (Linnavuori and DeLong 1979: figs 41–42).	***Parandanus cruciatus* Linnavuori & DeLong**

### Parandanus longistylus

Taxon classificationAnimaliaHemipteraCicadellidae

Duan sp. n.

http://zoobank.org/44D7A960-CB27-44BA-91C4-9D92189D469B

Figs 1 , 2

#### Description.

Length. Male: 6.8–7.2 mm.

**Figure 1.**
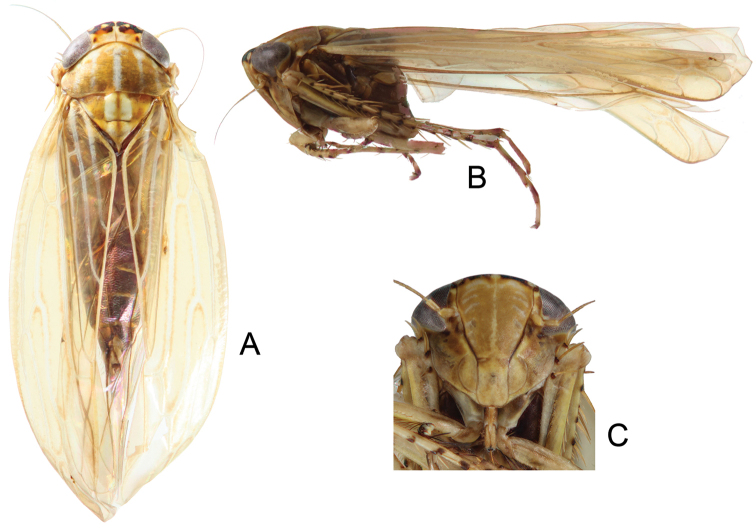
*Parandanus longistylus* Duan, sp. n. **A** habitus, dorsal view **B** habitus, lateral view **C** face.

Anterior margin of crown with two dark brown coalescing spots extending around ocelli, disk with an orange spot anterolaterally, its inner area dark brown, a small dark brown spot adjacent to basal angles of eyes. Pronotum with six broad longitudinal orange brown bands (Fig. 1A).

Crown of near uniform length, 0.43× as long as distance between eyes, 0.43× as long as median length of pronotum (Fig. 1A).

#### Male genitalia.

**Figure 2.**
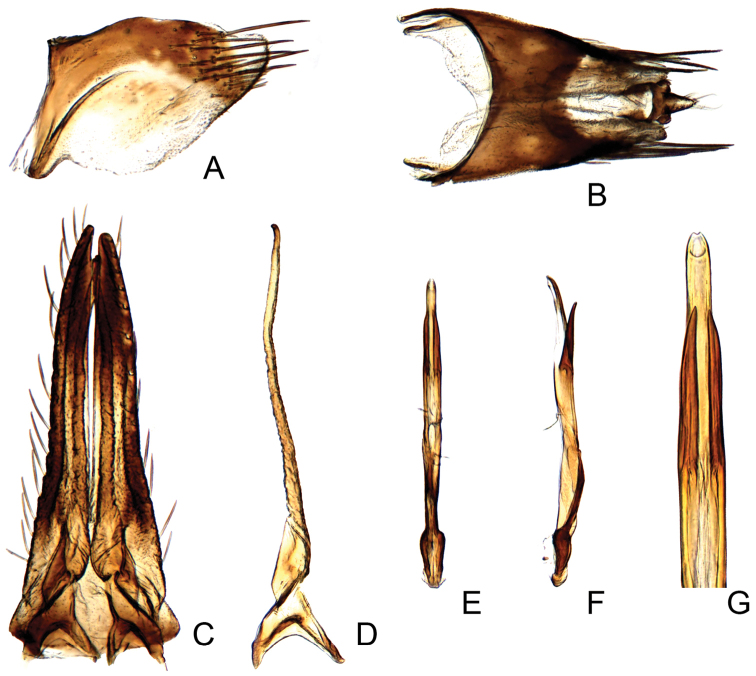
*Parandanus longistylus* Duan, sp. n. **A** male pygofer side, lateral view **B** male pygofer and segments X–XI, dorsal view **C** valve, subgenital plates and styles, ventral view **D** style, dorsal view **E, F** connective and aedeagus, dorsal and lateral view, respectively **G** apex of aedeagus, dorsal view.

Pygofer short, sides rounded apically (Fig. 2A–B). Subgenital plate elongate with lateral margins nearly straight, with approximately 17 narrow macrosetae (Fig. 2C). Style articulating arm short; preapical angle indistinct; apophysis very elongate (Fig. 2D). Aedeagus with pair of subbasal parallel appendages, extending to subapex of shaft (Fig. 2E–G).

#### Material examined.

Holotype: male, Peru: Pasco, 3km N Oxapampa, 1700m, 10°33.20'S, 75°25.55'W, 21 Oct 2002, C.H. Dietrich, merc vapor light, 02–31–1 (INHS). Paratypes: 3 males, same data as holotype.

#### Etymology.

This name is based on the style with a long apophysis.

#### Remarks.

This species is similar to *Parandanus paracruciatus* in color pattern but differs from this and other species by its very long style apophysis.

### Parandanus paracruciatus

Taxon classificationAnimaliaHemipteraCicadellidae

Duan sp. n.

http://zoobank.org/F13969FC-3F84-4092-AAEB-2CB70F7FCDE3

Figs 3 , 4

#### Description.

Length. Male: 6.6–7.0 mm.

**Figure 3.**
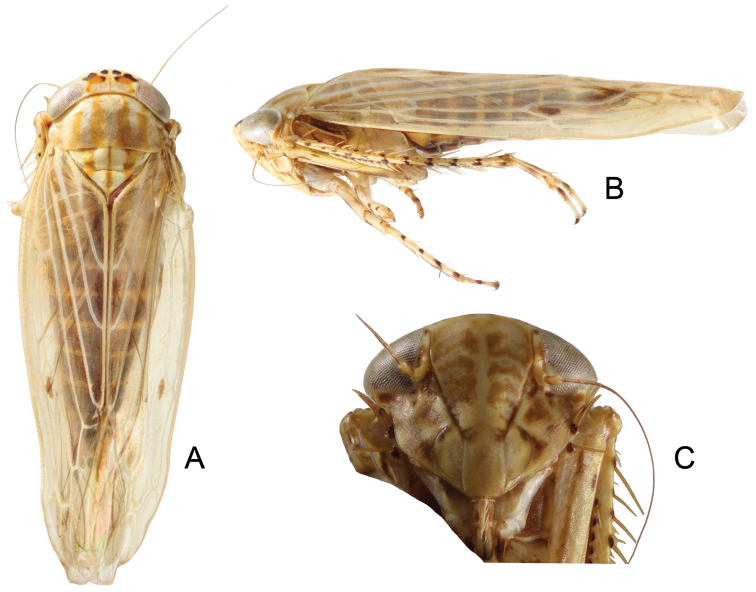
*Parandanus paracruciatus* Duan, sp. n. **A** habitus, dorsal view **B** habitus, lateral view **C** face.

Anterior margin of crown with four dark brown spots, disk with an orange spot laterally, its inner area dark brown, a small orange spot adjacent to basal angles of eyes. Pronotum with six narrow longitudinal orange brown bands (Fig. 3A).

Crown of nearly uniform length, 0.48x as long as distance between eyes, 0.47× as long as median length of pronotum (Fig. 3A).

#### Male genitalia.

**Figure 4.**
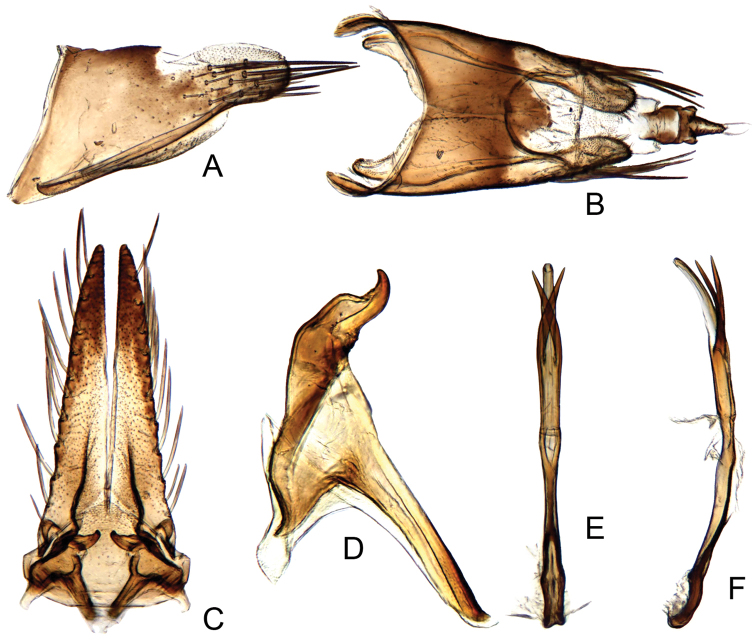
*Parandanus paracruciatus* Duan, sp. n. **A** male pygofer side, lateral view **B** male pygofer and segments X–XI, dorsal view **C** valve, subgenital plates and styles, ventral view **D** style, dorsal view **E, F** connective and aedeagus, dorsal and lateral view, respectively.

Pygofer long, sides rounded apically, ventral margin concave (Fig. 4A–B). Subgenital plate with lateral margins nearly straight, with approximately 12 narrow macrosetae (Fig. 4C). Style articulating arm very long; preapical angle distinct; apophysis very short (Fig. 4D). Aedeagus with pair of subbasal crossed appendages, extending to near to apex of shaft (Fig. 4E–F).

#### Material examined.

Holotype: male, Peru: Pasco, 3km N Oxapampa, 1700m, 10°33.20'S, 75°25.55'W, 21 Oct 2002, C.H. Dietrich, merc vapor light, 02–31–1 (INHS). Paratypes: 3 males, same data as holotype.

#### Etymology.

The species name is based on the similarity of the species to *Parandanus cruciatus*.

#### Remarks.

This species is similar to *Parandanus longistylus* in color pattern but differs from this and other species by the shape of the style with short apophysis and long articulating arm.

### Parandanus nigricephalus

Taxon classificationAnimaliaHemipteraCicadellidae

Duan sp. n.

http://zoobank.org/6EB41ED2-F47A-4CC0-AE6E-5B696F71C04C

Figs 5 , 6

#### Description.

Length. Male: 6.6–6.8 mm.

**Figure 5.**
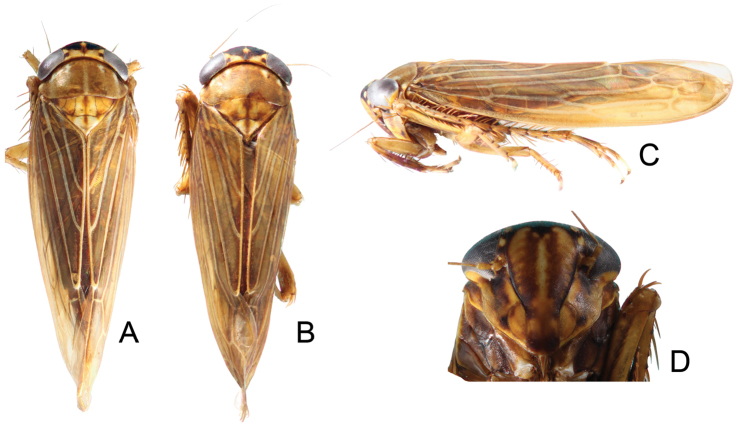
*Parandanus nigricephalus* Duan, sp. n. **A, B** habitus, dorsal view **C** habitus, lateral view **D** face.

Anterior margin of crown with a dark brown patch extending around ocellus and variably onto disc and to posterior margin, with a medial small yellow spot, a small dark brown spot adjacent to basal angles of eyes (Fig. 5A–B). Face with pronounced brown markings (Fig. 5D). Pronotum with longitudinal bands variably coalescing, sordid orange brown (Fig. 5A–B).

Crown of nearly uniform length, 0.36× as long as distance between eyes, 0.38× as long as median length of pronotum (Fig. 5A–B).

#### Male genitalia.

**Figure 6.**
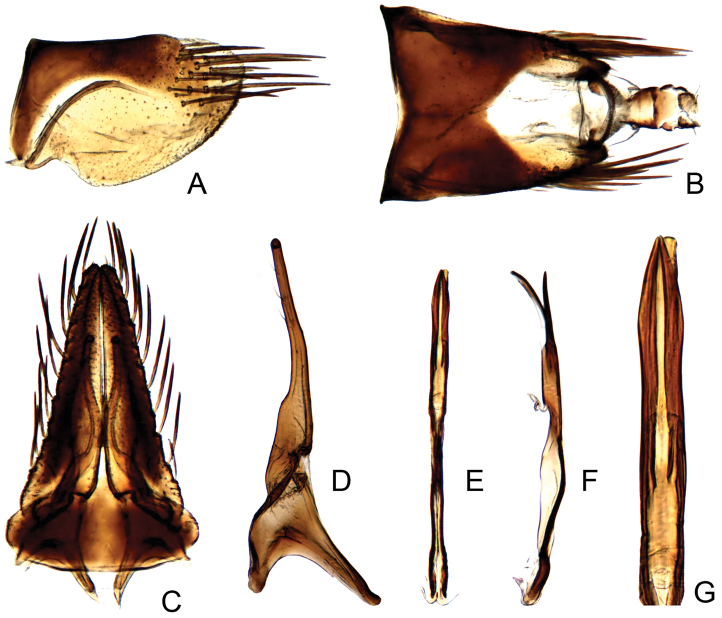
*Parandanus nigricephalus* Duan, sp. n. **A** male pygofer side, lateral view **B** male pygofer and segments X–XI, dorsal view **C** valve, subgenital plates and styles, ventral view **D** style, dorsal view **E, F** connective and aedeagus, dorsal and lateral view, respectively **G** apex of aedeagus, dorsal view.

Pygofer short, sides rounded apically (Fig. 6A–B). Subgenital plate with lateral margins nearly straight, with approximately 11 narrow macrosetae (Fig. 6C). Style articulating arm moderately long and robust; preapical angle indistinct; apophysis long (Fig. 6D). Aedeagus with pair of subbasal parallel appendages converging apically, extending near to apex of shaft (Fig. 6E–G).

#### Material examined.

Holotype: male, Peru: Pasco, 3km N Oxapampa, 1700m, 10°33.20'S, 75°25.55'W, 21 Oct 2002, C.H. Dietrich, merc vapor light, 02–31–1 (INHS). Paratypes: 13 males, same data as holotype.

#### Etymology.

This name is based on the vertex with a large dark patch.

#### Remarks.

This species can be distinguished by its pronounced brown head markings and long style apophysis.

### Parandanus hilaris

Taxon classificationAnimaliaHemipteraCicadellidae

Figs 7 , 8

Parandanus hilaris Linnavuori & DeLong, 1976: 34.

#### Description.

Length. Male: 5.5–5.9 mm.

**Figure 7.**
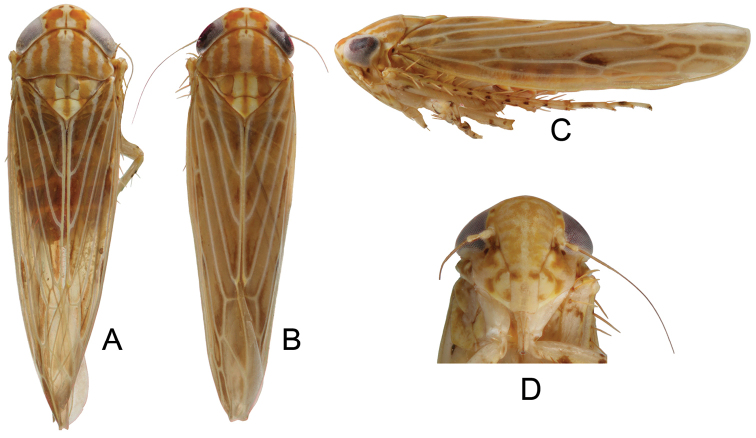
*Parandanus hilaris*. **A, B** habitus, dorsal view **C** habitus, lateral view **D** face.

Anterior margin of crown with six small brown spots, disk with orange patch on each side anteriorly and extending to posterior margin, a small brown spot adjacent basal angles of eyes. Pronotum with six longitudinal narrow orange bands (Fig. 7A–B).

Crown about 1.17× as long medially as next to eyes, 0.67× as long as distance between eyes, 0.56× as long as median length of pronotum (Fig. 7A–B).

#### Male genitalia.

**Figure 8.**
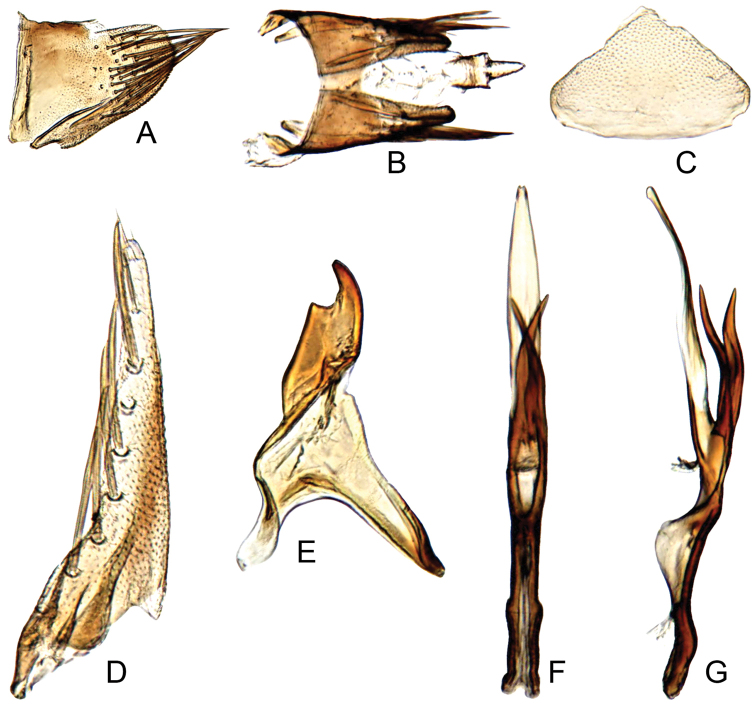
*Parandanus hilaris*. **A** male pygofer side, lateral view **B** male pygofer and segments X–XI, dorsal view **C** valve, ventral view **D** subgenital plate, ventral view **E** style, dorsal view **F, G** connective and aedeagus, dorsal and lateral view, respectively.

Pygofer short, sides rounded apically (Fig. 8A–B). Subgenital plate with lateral margins distinctly insinuated subbasally, with seven robust macrosetae (Fig. 8D). Style articulating arm moderately long and robust; preapical angle distinct; apophysis very short (Fig. 8E). Aedeagus with pair of relatively robust basal crossed appendages, sinuate in lateral view, extending to middle of shaft (Fig. 8F–G).

#### Material examined.

2 males, Peru: Huànuco, 5km S Tingo Maria, Pte. Perez, 600m, 9°20.51'S, 75°58.51'W, 25 Oct 2002, C.H. Dietrich, mercury vapor light, 02–41–1 (INHS); 1 male, Peru: Huànuco, 5km S Tingo Maria, Pte. Perez, 600m, 9°20.51'S, 75°58.51'W, 25 Oct 2002, R. A. Rakitov, mercury vapor light, 02–41–2 (INHS).

#### Distribution.

Peru.

#### Remarks.

This species can be distinguished by its relatively longer crown medially, few robust subgenital plate macrosetae, short syle apophysis and shape of the aedeagus.

## Supplementary Material

XML Treatment for Parandanus

XML Treatment for Parandanus longistylus

XML Treatment for Parandanus paracruciatus

XML Treatment for Parandanus nigricephalus

XML Treatment for Parandanus hilaris
